# Digital Support for Renal Patients Before and During the COVID-19 Pandemic: Examining the Efforts of Singapore Social Service Agencies in Facebook

**DOI:** 10.3389/fdata.2021.737507

**Published:** 2021-09-14

**Authors:** Junjie Tan, Aravind Sesagiri Raamkumar, Hwee Lin Wee

**Affiliations:** National University of Singapore, Singapore

**Keywords:** COVID-19, disease outbreaks, facebook, renal insufficiency, social media, social service agencies

## Abstract

During the coronavirus disease 2019 (COVID-19) pandemic, social service agencies (SSAs) play a crucial role in supporting renal patients, who are particularly vulnerable to infections. Social media platforms such as Facebook, serves as an effective medium for these SSAs to disseminate information. Content analysis of the SSAs’ Facebook posts can provide insights on whether Facebook has been adequately utilized during the COVID-19 pandemic and enable SSAs to improve their social media use in future pandemics. This study aimed to compare renal-related SSAs’ Facebook post content before and during the COVID-19 pandemic. Facebook posts of three SSAs National Kidney Foundation (NKF), Kidney Dialysis Foundation (KDF), and Muslim Kidney Action Association (MKAC), posted during the pre-COVID-19 period (January 23, 2019 to June 2, 2019) and the peri-COVID-19 period (January 23, 2020 to June 1, 2020) were extracted. A classification scheme was developed by two coders with themes derived inductively and deductively. Each Facebook post was assigned with a theme. Quantitative analyses indicate that the number of Facebook posts increased from 115 in the pre-COVID-19 period to 293 in the peri-COVID-19 period. During peri-COVID-19, posts regarding lifestyle changes, donations and infectious disease surfaced. While the proportion of posts about encouraging kindness increased from one to 77 posts, the proportion of posts about community-based events and psychosocial support decreased from 44 to 15 posts and 17 to 10 posts respectively during the two periods. Facebook was found to be well-utilized by two of the three renal SSAs in engaging their beneficiaries during the pandemic. During future pandemics, renal SSAs should place emphasis on posts related to psychosocial support and encouraging kindness. Further studies are required to ascertain the impact of COVID-19 from the perspective of renal patients and also to validate the classification scheme which was developed in this study. The study’s methodology and classification scheme can be used to guide future studies for evaluating the social media outreach performance of renal health support groups.

## Introduction

Social service agencies (SSAs) provide direct services to citizens at either public or private level in non-profit settings. These services are aimed at avoidance, improvement and resolution of issues pertaining to physical health, mental health, society and environment (Gibelman, 2005). In Singapore, SSAs are under the purview of National Council of Social Service (NCSS) which is a statutory board governed by the Ministry of Social and Family Development (National Council of Social Service, 2021). NCSS helps SSAs with funding, manpower and capacity building. At present, there are more than 400 SSAs in Singapore who are registered with NCSS (Directory of NCSS Members, 2021), providing support to the citizens for a range of issues. In light of the emergence of social media platforms, SSAs are encouraged to follow NCSS guidelines (MSF, 2021) and also engage with donors in such platforms (NCSS, 2019).

From the last decade, social workers who form the backbone of SSAs, have had the option of utilizing social media to engage with the beneficiaries (Westwood, 2019). Social media platforms can be mainly used to blend offline and online efforts in real-time (Sitter and Curnew, 2016), although ethical challenges of participating in such an online space have been identified (Chan, 2016; Boddy and Dominelli, 2017). In particular, Facebook was found to be beneficial for social workers in the context of informal peer-support and emotional support (Gandy-Guedes et al., 2016). Another study showed that social workers get drawn to Facebook to know more about the beneficiaries while they are attempting to provide better service (Cooner et al., 2020). Apart from Facebook, Twitter and YouTube appear to be the other most frequently used social media platforms (Young, 2016). Studies have been conducted to understand the usage intents of social service organizations in Facebook and Twitter. Although both platforms are used predominantly for information sharing, Twitter gets used more for requesting their followers to take actions towards health betterment (Campbell and Lambright, 2020). Nonetheless, familiarity with social media platforms is bound to help those involved in social service (Fagan et al., 2018).

The coronavirus disease 2019 (COVID-19) was declared a pandemic by the World Health Organization (WHO) on March 11, 2020 (World Health Organization, 2020). Patients with renal disease are particularly vulnerable and more likely to have poorer clinical outcomes if infected with COVID-19 (ADVISORY ON VULNERABLE GROUP, 2020; Khoo et al., 2020). As such, they may be experiencing considerable anxiety. This situation is alarming because Singapore has approximately one new case of kidney failure every 5 hours (National Kidney Foundation, 2020a). Furthermore, the number of dialysis patients in Singapore was 7,405 in 2018 and has been increasing since 2009 (Khor et al., 2020). In response to COVID-19, community dialysis centres have implemented telemedicine (Lee Ying Ngoh et al., 2020) and adopted additional measures to mitigate infection risks (Khoo et al., 2020). Given that hospital services are mostly postponed (Khalik, 2020), SSAs in Singapore play a crucial role in supporting this group of patients. SSAs and social workers are expected to play an important role in the control of COVID-19 spread in the community (Amadasun, 2020).

During the ongoing COVID-19 pandemic, more web-based information needs to be made available for patients who require renal transplantation (van Klaveren et al., 2020). It has been well-established that social media plays an immense role in disaster communication and risk communication since it enables new pathways of information seeking and sharing, and exchanges of assistance (Palen and Hughes, 2018). Before the COVID-19 pandemic, social media platforms have been found to be well-used for prior epidemics such as Zika, H1N1 and Dengue (de Araújo et al., 2020). In the midst of a pandemic, with safe distancing measures in place, social media may be the only viable channel, other than mainstream media and online websites, for disseminating information and, hence, it should be put to good use (Sharma et al., 2017; Chan et al., 2020; Lima et al., 2020). The benefits of social media have been well identified for urological community (Shah and Davies, 2020). Increasingly, patients are using social media to look for health information (Chretien and Kind, 2013) and this further emphasizes the role of social media as a useful communication tool. On the other hand, studies have been conducted to ascertain the nature and quality of communication in social media platforms. An analysis of health-related Facebook posts/tweets indicated that the communication largely remained one-way with minimal interaction albeit the need for increasing the user base in such platforms has been raised (Huang and Dunbar, 2013). From the patient’s perspective, a lack of clarity was observed with the health messages posted in social media platforms along with lack of engagement (De Las Heras-Pedrosa et al., 2020). With health-related social media communication still being in its infancy, there is a need to develop a social media posting guide for healthcare providers so that patients are benefitted during both normal and pandemic situations.

Renal disease management is critical for patients since early identification can help in slowing down the disease progression and reducing further complications, thereby facilitating a smooth transition to appropriate therapies (Rastogi et al., 2008). Moreover, renal patients are prone to neuropsychiatric conditions (Simões e Silva et al., 2019), hence disease management support through social service organizations is much needed. As renal SSAs assist beneficiaries in providing affordable and quality education along with support for kidney disease prevention, their activities need to be understood for research and policy making purposes. Currently, no study has been conducted to evaluate the use of social media by SSAs to support patients with renal disease during the COVID-19 pandemic. To examine Facebook pages of health agencies, previous studies have utilized content analysis (Janice, 2019; Sesagiri Raamkumar et al., 2020). Hence, a content analysis of the SSAs’ Facebook posts may provide insights on how Facebook is utilized during the COVID-19 pandemic. Furthermore, this analysis may enable SSAs to improve their usage of social media to reach out to patients with renal disease during future pandemics.

The aim of this study was to analyze the content of Facebook posts published by renal SSAs before and during the COVID‐19 pandemic. We focused specifically on Facebook since it is the most popular social media platform in Singapore (Kemp, 2020). The SSAs to be analyzed in this study were selected from the National Council of Social Services’ list of members (Directory of NCSS Members, 2021). Three SSAs that have patients with renal disease as their main target audience are 1) National Kidney Foundation (NKF), 2) Kidney Dialysis Foundation (KDF) and 3) Muslim Kidney Action Association (MKAC). Accordingly, these three SSAs were selected for the current study. NKF has been supporting renal patients for more than 50 years and it has been using Facebook for outreach through its official page since 2012 (National Kidney Foundation, 2020b). KDF has been supporting renal patients in acquiring funds for dialysis since 1996 and they have been posting content in Facebook from 2009 (Kidney Dialysis Foundation, 2020). The MKAC was established to support chronically ill renal patients since 2004 and the corresponding Facebook page was launched by them in 2011 (Muslim Kidney Action Association, 2020).

We sought to address three objectives pertaining to the three SSAs’ Facebook use. First, we sought to determine the posting frequency of the three SSAs in Facebook before and during the COVID-19 pandemic. The purpose was to gain insights on any changes in activity level of the Facebook pages. The second objective was to develop a classification scheme for the SSAs’ Facebook posts. The third objective was to use the classification scheme for performing content analysis of Facebook posts. This will enable us to analyze how the contents posted by the three SSAs have changed over time. The classification scheme provides the scope to be re-used validated in future studies while evaluating social media content of any type of health related SSAs.

## Methods

### Data Extraction

Facebook data was extracted using Facepager (Jünger and Keyling, 2019). Data from the three SSAs were extracted on July 28, 2020. On that given day, the number of followers of NKF (National Kidney Foundation, 2020b), KDF (Kidney Dialysis Foundation, 2020) and MKAC (Muslim Kidney Action Association, 2020) were 15,862, 4,658 and 874, respectively. Data of Facebook posts were extracted for two time periods namely peri-COVID-19 and pre-COVID-19. The peri-COVID-19 period was set from January 23, 2020 to June 1, 2020. The pre-COVID-19 period was set from January 23, 2019 to June 2, 2019. January 23, 2020 was selected as the start of the peri-COVID-19 period as the first COVID-19 patient was diagnosed on this date in Singapore (Goh and Wei, 2020). On the other hand, June 1, 2020 was selected as the end of the peri-COVID-19 period because the circuit breaker (i.e. lockdown) in Singapore ended on this date (gov.sg, 2020a). For ease of comparison, the start date for the pre-COVID-19 period was selected as exactly 1 year before that of the peri-COVID-19 period. Additionally, the end date of the pre-COVID-19 period was 1 day after that of the peri-COVID-19 period since 2020 is a leap year.

### Exclusion Criteria

A total of 14 Facebook posts without any text messages were excluded from the analysis. Eight non-English posts, which did not contain any direct English translations, were also excluded. Content of videos were not included in analysis and only their corresponding caption was analyzed. Additionally, contents of images were not analyzed unless themes could not be identified from the corresponding text without them.

### Identification of Themes

For identification of themes, content analysis of Facebook posts was carried out according to recommendations from Wimmer and Dominick (2015) and Lacy et al. (2015). Construction of content themes was achieved both inductively and deductively, in the following manner. Themes were derived inductively via emergent coding for which a preliminary examination of data was conducted. Additionally, some of the themes were also derived deductively from the list of content for consumers on the National Kidney disease Education Program (NKDEP) website (Goldstein et al., 2013). The development of classification scheme was performed by two coders (first author and research team member). The initial classification scheme was constructed by the primary coder (first author). Subsequent revisions to the scheme were made after two pilot studies, during which the primary and secondary coder discussed on the theme assignment heuristics until a consensus was reached. Fifty randomly selected posts were coded during each pilot study. Subsequently, using the finalized classification scheme, both coders independently coded 106 randomly selected posts and intercoder reliability was calculated. An acceptable Krippendorff’s alpha (α) value of 0.9 was achieved through this process. Since an acceptable level of intercoder reliability was achieved, the primary coder proceeded to assign themes for all 408 study posts.

Using the classification scheme, each post was assigned to a theme. If two themes were present in the same post, the theme which represented the Facebook post in a major way was assigned as the theme. Facebook posts that did not fall under any of the constructed themes were labelled as *Others*. To achieve exhaustivity and avoid missing relevant themes, we re-examined and revised the posts in the *Others* category so that the proportion of posts that fell into this category was 10% or below (Wimmer and Dominick, 2015). If the number of posts under the *Others* category exceeded 10%, the classification scheme was re-examined and revised. More details about the content analysis are available in Supplementary Appendix A of the supplementary file. The content of the Facebook posts and the corresponding themes have been made publicly available (Tan et al., 2021).

### Analysis Procedures

For comparison of number of posts between the pre-COVID-19 and peri-COVID-19 period for each SSA, Poisson regression was used. Similarly, Poisson regression was used to compare the number of posts assigned to a particular theme between the pre-COVID-19 and peri-COVID-19 period. To compare the proportion of posts assigned to a particular theme between the pre-COVID-19 and peri-COVID-19 period for each theme, the Chi-square test and Fisher’s exact test were used. A significance level of 0.05 was set for this study. The intercoder reliability coefficient was reported as Krippendorff’s alpha (Krippendorff, 2011), as recommended by Wimmer and Dominick (2015) and Lacy et al. (2015), with an acceptable level set at 0.8 and above.

## Results

### Social Service Agencies’ Posts in Facebook

**FIGURE 1:**
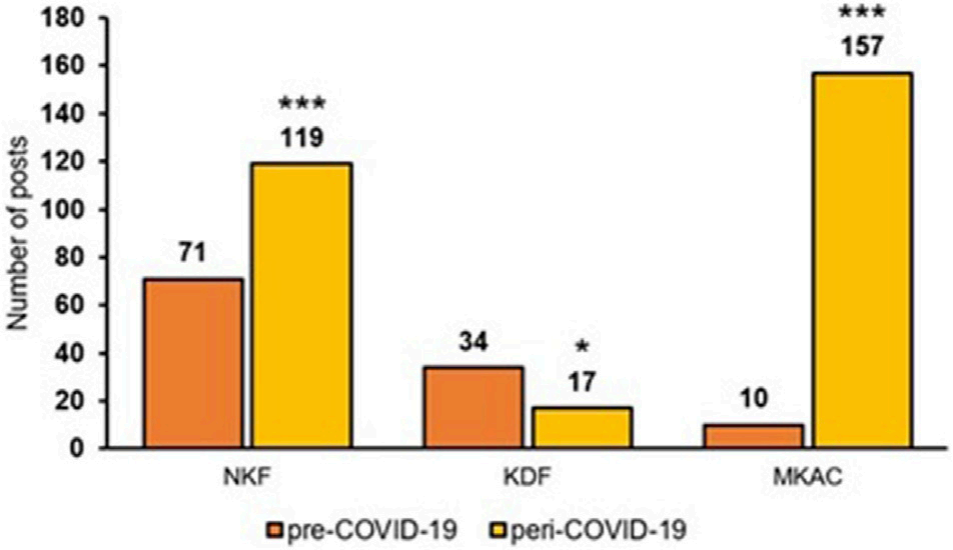
Number of posts by the SSAs in pre-COVID-19 and peri-COVID-19. *: *p*-value ≤ 0.05; ***: *p*-value ≤ 0.001.

**FIGURE 2:**
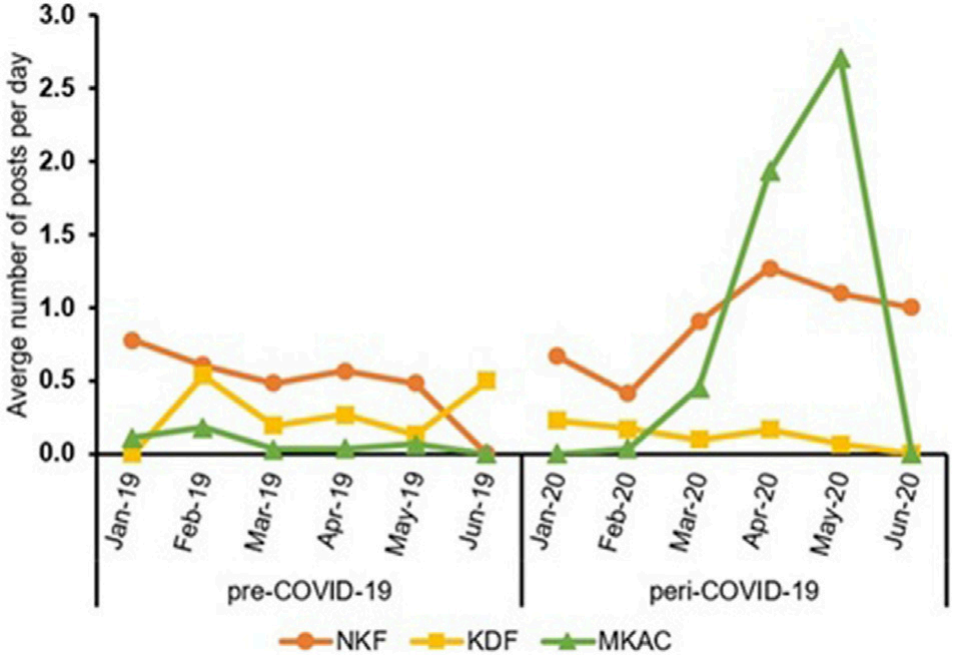
Average number of posts per day by the three SSAs for each month during pre-COVID-19 and peri- COVID-19 periods.

Figure 1 shows the number of Facebook posts from NKF, KDF, and MKAC during the pre-COVID-19 and peri-COVID-19 periods. Compared to the pre-COVID-19 period, the number of posts were higher during the peri-COVID-19 period for NKF and MKAC but not for KDF. Figure 2 shows the monthly average number of posts per day during the pre-COVID-19 and peri-COVID-19 periods. The daily posting frequency of the three SSAs was relatively consistent for all 6 months during the pre-COVID-19 period. However, the daily posting frequency of NKF and MKAC increased considerably in the month of March, April and May 2020 during the peri-COVID-19 period.

### Themes of SSAs’ Facebook Posts

The classification scheme consists of ten themes, excluding the Others theme. Four themes were derived from the NKDEP’s content for consumers: 1) Lifestyle changes, 2) Nutrition education, 3) CKD and 4) Healthcare team and programmes. The six remaining themes were: 1) Donations, 2) Community-based events, 3) Appreciation, 4) Infectious disease, 5) Psychosocial support and 6) Encouraging kindness. Descriptions of each theme with sample representative verbatim quotes are presented in Table 1. The number and proportion of posts for each theme during the pre-COVID-19 and peri-COVID-19 period are listed in Table 2. Representative verbatim quotes for the ten themes are listed in Supplementary Appendix B.

**TABLE 1:** Descriptions of themes in the Facebook posts.

Theme	Descriptions of posts representing the theme	Representative verbatim quotes
Lifestyle changes	Contains information about physical activity, weight management or smoking cessation	“Here’s another intermediate workout video with variation for you to choose from based on your fitness level.”
	Encourages physical activity, weight management or smoking cessation	“Exercise for at least 30 min, at moderate intensity, five times a week and achieve a healthier lifestyle!”
Nutrition education	Provides recommendations for dietary or fluid intake	“#DidYouKnow Curry Fish has 234 calories, 1,254 mg sodium and 12 g of fat. Assam Fish has 123 calories, 532 mg sodium and 3.7 g of fat. The healthier choice here is Assam Fish, as it has significantly lower caloric value, sodium and fat content.”
	Encourages healthy eating	“We hope that you have learnt a thing or two with us through these healthy tips and be able to eat better and wisely, while choosing the right food for your body!”
	Sharing of healthy recipes	“Try out our version of a healthier kidney-friendly egg omelette with tuna, ready in 10 min for an afternoon snack!
CKD	Contains information about	“Fluid overload can affect you, from shortness of breath to the swelling of feet, ankles and face.”
	• Symptoms of CKD (chronic kidney diseases)	“Hypertension, also known as High Blood Pressure is the second leading cause of kidney failure in Singapore? It is often called the ‘silent killer’, because it does not always show symptoms and 1 in 4 Singaporean adults have hypertension.”
	• Complications of CKD	“Patients with kidney disease need to maintain a normal blood pressure and the use of specific blood pressure agents (one of two drug groups known as ACE–inhibitors and ARB agents) help to protect the kidney against damage.”
	• Causes of CKD (e.g., hypertension, diabetes)	
	• Treatment of CKD (e.g., dialysis, kidney transplant, medications)	
Healthcare team and programmes	Contains information about the job scope of healthcare professionals	“Have you ever wondered how is it actually like to work as a nurse? There are no typical days if you are a nurse. Meet XYZ, our NKF nurse to find out a little more”
	Contains information or promotes services available for CKD patients (e.g., dialysis, consultation with healthcare professionals)	“With the opening of The Hour Glass-NKF Dialysis Centre at West Coast, kidney patients like XYZ, who were previously receiving treatment at other dialysis centres further away from their homes, now enjoy greater convenience and easier access to dialysis care.”
	Contains information or promotes activities which are organized solely for CKD patients (e.g., health talks, crafts)	“MKAC held its fourth batik painting session. With the guidance of batik instructor, XYZ, our kidney patients expressed themselves on the stretched fabric”
Donations	Requests for donations	“please extend your assistance by making a donation at: www.xyz.com
		“Contribute through PayNow to UEN: AAA”
Community-based events	Contains information or promotes events organized for the general public (e.g., fund-raising events, charity, kidney health screening, carnivals, walkathons, online contests, online events)	“Just 3 days to go before our online charity auction closes on June 3, 2020 12 midnight.”
		“Comment in the box below and stand a chance to win $10 NKF NETS Flashpay Card!”
		“KDF’s World Kidney Day Carnival 2019 is open to the public, so bring your friends and family!”
Appreciation	Expresses gratitude to either healthcare workers, volunteers, caregivers, kidney donors, sponsors or the general public	“A heartfelt thank you to all healthcare staff for your service to patients.”
	Promotes events which allow others to show their gratitude	“Send in your messages and appreciation here www.xyz.com, and we will convey them for you!”
	Mentions celebrations (e.g., Nurses’ Day)	“Happy International Nurses Day to all of our nurses!”
Infectious disease	Contains information about symptoms and complications of infectious diseases	“Here’s an interesting read on how this virus can cast a storm to all the organs, including kidneys too!”
	Encourages good hygiene practices	“Here are some steps you should take to protect yourself”
	Sharing of news related to infectious diseases	
Psychosocial support	Sharing of patients’, caregivers’, or kidney donors’ experiences	“XYZ who has been on dialysis for the past 40 years, is also Singapore’s longest surviving patient on dialysis. Here is his story”
	Use of motivational quotes	“Never bend your head. Always hold it high. Look the world straight in the eye.”
	Provides tips for better mental well-being	“The stress of being constrained with stressed family members can be very challenging, making family relationships more tense than ever. Click on the images and learn a few tips to get you through from our Clinical Psychologist, XYZ.”
Encouraging kindness	Sharing of kindness quotes	“A kind gesture can mean the world to someone.”
		“A warm, supportive home that brings joy to the family members.”

**TABLE 2:** Summary of assignment of posts to the themes.

Theme	Pre-COVID-19 (n = 115), n (%)	Peri-COVID-19 (n = 293), n (%)	p^a^	p^b^
Lifestyle changes	0 (0.0)	12 (4.1)	NA^c^	0.023
Nutrition education	12 (10.4)	27 (9.2)	0.019	0.710
CKD	10 (8.7)	19 (6.5)	0.100	0.520
Healthcare team and programmes	7 (6.1)	10 (3.4)	0.469	0.270
Donations	0 (0.0)	55 (18.8)	NA^c^	<0.001
Events^d^	44 (38.3)	15 (5.1)	<0.001	<0.001
Appreciation	13 (11.3)	23 (7.8)	0.100	0.331
Infectious disease	0 (0.0)	23 (7.8)	NA^c^	0.003
Psychosocial support	17 (14.8)	10 (3.4)	0.183	<0.001
Encouraging kindness	1 (0.9)	77 (26.3)	<0.001	<0.001
Others	11 (9.6)	22 (7.5)	NA^e^	NA^e^

a *p*-value of Poisson regression (for counts comparison).

b *p*-value of Chi-square test or Fisher’s exact test (for proportions comparison).

c NA: *p*-value cannot be computed when one of the counts is zero.

d Events: Community-based events theme.

e NA: *p*-value not computed for Others theme.

As seen from Table 2, three new themes *Lifestyle changes, Donations* and *Infectious disease* emerged during the peri-COVID-19 period. Additionally, the number of posts assigned to *Nutrition education* and *Encouraging kindness* increased significantly. However, there was no significant difference in the proportion of posts assigned to *Nutrition education* between both periods. Within the theme *Nutrition education*, the number of posts focusing on cooking at home was higher for the peri-COVID-19 period (n = 14) as compared to the pre-COVID-19 period (n = 3). On the other hand, during the peri-COVID-19 period, the number of posts assigned to *Events* decreased significantly. For the theme *Psychosocial support*, while no significant difference in the number of posts was observed, the proportion of posts decreased significantly.

## Discussion

During the peri-COVID-19 period, the activity level of NKF’s and MKAC’s Facebook page increased significantly, as indicated by the spike in number of posts during the months of March, April, and May 2020. This spike coincides with the declaration of COVID-19 as a pandemic by the WHO in March 2020 (World Health Organization, 2020) and the start of the circuit breaker (i.e. lockdown) in April 2020 (gov.sg, 2020b). Consequently, the two SSAs increased their outreach efforts through Facebook. The increased frequency of posting can be regarded as desirable, considering that social media has the potential to be used as an effective communication tool during a pandemic (Sharma et al., 2017; Chan et al., 2020; Lima et al., 2020). Interestingly, the posting frequency of KDF’s Facebook page dropped during the peri-COVID-19 period. More than half of the posts made by KDF during pre-COVID-19 period were assigned to the *Community-based events* theme. As such, with social distancing measures put in place during the pandemic, a lack of physical events could have led to the drop in number of posts in KDF’s Facebook page. Another possible reason for the drop in the posting frequency could be that the team managing KDF’s Facebook page was diverted to support the other functions of KDF.

During the peri-COVID-19 period, SSAs began to post about *Lifestyle changes, Donations* and *Infectious disease*. Posts on *Lifestyle changes* are appropriate during a pandemic as lockdowns may discourage people from leading an active lifestyle (Ricci et al., 2020). While lifestyle changes could include smoking cessation, all 12 study posts assigned to *Lifestyle changes* were related to physical activity. This observation probably reflects the concern that patients with renal disease were spending less time outdoors. The strategy of posting about *Lifestyle changes* is also in line with the content available for consumers on the National Institute of Diabetes and Digestive and Kidney Diseases (NIDDK) website (NIDDK, 2016a). Posts related to *Donations* surfaced during the COVID-19. The lockdown had prevented the SSAs from conducting physical fundraising activities (Lim, 2020). To address this limitation, the SSAs shifted their fundraising efforts online, making use of their social media platforms to request for donations. However, we were currently unable to determine if the SSAs’ online fundraising efforts yielded comparable results as their usual offline fundraising activities. As such, the usefulness of such posts could not be established. Nevertheless, online fundraising is probably the only viable alternative to physical fundraising activities during a pandemic.

The last new theme which emerged during the COVID-19 period was *Infectious disease*. Considering that Singapore was in the middle of a pandemic, this theme was expected. It has been suggested that community organizations play a crucial role in ensuring the dissemination of accurate information during a pandemic due to the surge in amount of misinformation (Tasnim et al., 2020). Hence, it is encouraging to see the renal SSAs posting content related to COVID-19 for increasing the awareness of this new infectious disease among their beneficiaries. Additionally, posts encouraging compliance to infection prevention measures is considered beneficial during the COVID-19 pandemic since patients with renal disease are particularly vulnerable to infections (ADVISORY ON VULNERABLE GROUP, 2020).

In addition to the emergence of the three new themes, it was found that the number of posts related to *Nutrition education* and *Encouraging kindness* increased during the COVID-19 period. Dietary education about sugar and salt intake is crucial since hypertension and diabetes are the two main causes of CKD (Drawz and Rahman, 2015). It is also part of the content available for consumers on the NIDDK’s website (NIDDK, 2016b). However, all 39 posts assigned to this theme were published by NKF. It is unusual that the other two SSAs did not post contents related to nutrition. Interestingly, more posts focused on cooking at home during the peri-COVID-19 period and this posting behavior could be attributed to the fact that people were expected to stay at home, particularly during the circuit breaker. This finding shows that the SSAs adapted their content to the pandemic situation and it can be a good learning point for future pandemics. For the theme *Encouraging kindness*, the number of posts during the COVID-19 pandemic increased significantly. The increase could be in response to the rise in cases of domestic violence during the circuit breaker in Singapore (Jean, 2020). Around the world, many other countries are facing this issue too (Bradbury-Jones and Isham, 2020). Staying at home may strain relationship among family members and result in violent behaviors. As such, amidst the pandemic, it is prudent for SSAs to promote kindness and encourage tolerance. Another possible reason why the SSAs posted more about *Encouraging kindness* could be their intention to encourage donations and generosity.

Contrary to our assumption, the SSAs posted less regularly about *Psychosocial support*. The COVID-19 pandemic has impacted psychosocial health negatively, causing increased fear and anxiety (Dubey et al., 2020). Thus, it is prudent for SSAs to provide social support to reduce psychological stress (Siegal et al., 1987). Despite the drop in proportion of *Psychosocial support* posts during the COVID-19 period, a shift from sharing of patients’ experience to sharing of stress management tips was observed. This is especially helpful for patients with renal disease and should be incorporated into SSAs’ response to future pandemics. The number of posts related *to the theme Community-based events* dropped in peri-COVID-19 period. This drop is understandable due to the social distancing measures put in place, limiting the number of events organized. We also found a increase in the number of online activities during the COVID-19 pandemic and this is a good way for the SSAs to engage their audience. For instance, online giveaways and contests were conducted. Hence, these events hold great potential to be used for education about renal health and the COVID-19 infection.

There are certain limitations in this study. Only two independent coders were involved in the assignment of themes for the posts. Having three or more coders would be more desirable to identify sources of disagreements (Lacy et al., 2015). However, due to the time-consuming nature of content analysis, it is not uncommon for published studies to use two independent coders (Lacy et al., 2015). The second limitation of this study was that we only focused on the SSAs’ Facebook pages. However, we understand that the three SSAs also have a social media presence on Instagram. Hence, our analysis may not fully capture the SSAs’ social media activity. Despite this limitation, we were also aware that KDF and MKAC did not have any Instagram posts during the pre-COVID-19 period and this meant that comparison of Instagram posts in both periods were not possible. It is to be noted that only one theme was assigned to each post during the theme assignment exercise. Although, the coders identified the major theme in the posts, there could be a few posts with multiple themes. However, we do not expect the lack of data on such posts to impact the study findings as their presence is expected to be miniscule. Lastly, we excluded non-English posts. This might have led to a loss of data and could have affected our findings. However, due to the tedious nature of translating qualitative research data (Regmi et al., 2010) and the small proportion of non-English posts, excluding them was deemed to be an acceptable alternative.

In summary, Facebook was considerably well-utilized by two out of three SSAs during the COVID-19 pandemic. The SSAs posted more regularly and covered relevant topics in response to the COVID-19 pandemic. Considering our study findings, we recommend that in future pandemics, agencies should post about lifestyle changes, nutrition education, information about the infection and stress management tips. Additionally, agencies should aim to spread kindness and provide psychosocial support. They should also adapt their posts according to the pandemic, vary their post content and consider incorporating education into online giveaways and contests.

## Conclusion

Facebook was found to be a valuable platform for renal SSAs to engage their beneficiaries, who are particularly vulnerable to infections, during a pandemic. Through this study, we investigated the effects of the COVID-19 pandemic on the activity level of the three Singapore SSAs’ Facebook pages. Overall, our results showed that the two of the three SSAs posted more regularly after the onset of COVID-19 pandemic, suggesting their shift in outreach efforts toward social media. Additionally, we constructed a classification scheme. All the categories from this scheme provide the scope to be adopted in social media studies focusing on other health conditions. Nevertheless, this scheme needs to be validated with data from other countries to make it more generalizable. Through our content analysis of Facebook posts, we identified three new themes which emerged because of the COVID-19 pandemic, indicating that Facebook was rather well-utilized by the SSAs. Our findings also highlight the importance of certain themes of posts during a pandemic. Specifically, renal SSAs should place emphasis on posts related to lifestyle changes, nutrition education, information about the infection and stress management tips, during future pandemics. Moreover, SSAs should aim to spread kindness and provide psychosocial support through their social media platform.

One of the strengths of this study was that the two time periods of analysis had similar duration, thereby facilitating the comparison of results. Another strong point was that the entire set of Facebook posts were analyzed during both time periods of analysis, instead of analyzing only a sample of Facebook posts. In addition, we developed a novel classification scheme and took steps to ensure its reliability. The classification scheme could be used by SSAs, hospitals, public health authorities (PHAs) and other health support groups to post relevant content in social media platforms. Lastly, this study is the first study to our knowledge, to analyze social media content of renal SSAs during the COVID-19 pandemic. Moving forward, future studies can consider looking at SSAs which are supporting other vulnerable groups of patients, such as patients with chronic heart disease. Future studies can validate the classification scheme developed in this study, using data from renal-related SSAs in other countries.

## Data Availability

The dataset is available at this link https://doi.org/10.6084/m9.figshare.15185826.v2.
