# Supplementary material for: Digital Support for Renal Patients Before and During the COVID-19 Pandemic: Examining the Efforts of Singapore Social Service Agencies in Facebook
Source: Front Big Data. 2021 Sep 14;4:737507. doi: 10.3389/fdata.2021.737507 (PMC8476881; doi:10.3389/fdata.2021.737507)
Supplement: Supplementary file 1 [file DataSheet1.pdf]

Supplementary Material

Appendix A - Content Analysis Procedure

Two pilot studies involving 50 randomly selected study posts from the three SSAs was conducted to ensure intercoder reliability and to refine the classification scheme. Training sessions, where non-study posts extracted from the three SSAs' Facebook pages were examined and coded, were conducted for one secondary coder by the primary coder. Both coders had access to the full classification scheme, accompanied by descriptions for each theme. After the training sessions were completed, themes were entered by the two coders independently into a standardized Excel sheet. If an acceptable level of intercoder reliability was not achieved, both coders developed heuristics to assign themes to posts. If otherwise, the primary coder proceeded to assign themes for all 408 study posts.

Next, an intercoder reliability check, involving the secondary coder coding a reliability sample, was carried out to determine an intercoder reliability coefficient. The reliability sample was randomly selected from all the study posts. Sample size was calculated to be 106 posts based on a formula developed by Lacy et al [56]. The secondary coder independently assigned themes for the reliability sample. Subsequently, the intercoder reliability coefficient was calculated to ensure that reliability was at an acceptable level. Disagreements were resolved between the two coders via discussions [40]. After reaching a consensus for all posts in the reliability sample, themes assigned to the posts were finalized. Intracoder reliability was not checked since the coding process did not last beyond one month [40].

In Table A, a summary of the results of the two pilot studies and the reliability sample are listed. As seen in Table A, although the first pilot study achieved an acceptable level of intercoder reliability (Krippendorff's alpha $\geq$ 0.8), the proportion of posts labelled as Others exceeded 10%. Hence, we added an additional theme, Community-based events, to encompass some of the posts assigned to the Others theme. To improve reliability and replicability of the classification scheme, inclusion and exclusion criteria were included. After revision of the classification scheme, the second pilot study yielded an acceptable level of intercoder reliability and an acceptable proportion of posts under the Others theme. Similarly, the reliability sample achieved acceptable results. To achieve these levels of reliability, the number of training hours for the first and second pilot study were 2 hours and 1 hour, respectively.

Table A Summary of intercoder reliability tests conducted

Study/Sample	Agreements, n (%)	Alpha[a]	Others theme[b], n (%)
Pilot study 1 (n=50)	45 (90.0)	0.8813	11 (22.0)
Pilot study 2 (n=50)	46 (92.0)	0.9088	5 (10.0)
Reliability sample (n=106)	99 (93.4)	0.9256	10 (9.4)
